# Assessing hard and loose “endpoints”: comparison of patient and expert Bristol Stool Scale scoring of 2280 fecal samples

**DOI:** 10.12688/f1000research.152496.1

**Published:** 2024-07-25

**Authors:** Hanna Fjeldheim Dale, Milada Hagen, Gunn Helen Malmstrøm, Jennifer T. Fiennes, Marte Lie Høivik, Vendel A. Kristensen, Jørgen Valeur

**Affiliations:** 1 Department of Clinical Support, Lovisenberg Diaconal Hospital, Oslo, Norway; 2 Unger-Vetlesen Institute, Lovisenberg Diaconal Hospital, Oslo, Oslo, Norway; 3 Research Department, Lovisenberg Diaconal Hospital, Oslo, Norway; 4 Faculty of Health Science, Oslo Metropolitan University, Oslo, Norway; 5 Department of Gastroenterology, Oslo University Hospital, Oslo, Norway; 6 Institute of Clinical Medicine, University of Oslo, Oslo, Norway

**Keywords:** Fecal sample, stool form assessment, diarrhea, constipation

## Abstract

**Background:**

Stool consistency is an important outcome measure to evaluate in the investigation of several gastrointestinal diseases. The Bristol Stool Scale (BSS) is one of the most commonly used tools for evaluation of stool consistency. BSS ranges from 1-7 and each score is assigned to a given consistency of the feces. Self-reported characterizations can differ from an expert evaluation, and the reliability of BSS is unclear. We aimed to evaluate the reliability of BSS by comparing patient scores with expert scores.

**Methods:**

Patients with inflammatory bowel disease collected stool samples throughout a 3-year follow-up. The stool´s consistency was evaluated with BSS by the patients and matched with an expert score. Agreement between patient and expert scores was assessed using Cohen’s kappa.

**Results:**

BSS scores from 2280 fecal samples collected from 992 patients at up to five time points were included. When all samples were compared, there was good to substantial agreement between patient and expert scores (Cohen’s weighted kappa: 0.66-0.72). When the BSS scores were simplified and categorized as 1 (scores 1-2), 2 (scores 3-5) or 3 (scores 6-7), the agreement improved slightly (Cohen’s weighted kappa: 0.73-0.77). When the scores from the first sample per patient were compared, the experts were more likely to assign higher scores compared to the patient. The proportion of the lowest assigned scores (1-2) was 12.1% for patients and 8.1% for experts.

**Conclusions:**

The agreement between patient and expert BSS scores is good to substantial, especially when the BSS scores are simplified into three categories.

## Introduction

Stool consistency is a central component in the description of bowel habits and an important outcome measure to evaluate in the investigation of several gastrointestinal (GI) diseases. ^1^ Altered bowel habits are often seen as a consequence of diseases in the GI tract, and the water content in the stools affecting the consistency can reflect the intestinal transit time. Rapid intestinal transit time limits the absorption of water and cause loose or liquid stools (diarrhea), whereas slow intestinal transit causes extensive water absorption and harder stools (constipation). ^2^ The exact evaluation of stool consistency can be made by measurement of viscosity and stool water content, however this method requires cumbersome laboratory analyses and is not well suited for routine purposes. The form of the stool can serve as a proxy measure for the stool consistency, referring to the visually assessed shape and texture. ^3^

The Bristol Stool Scale (BSS) is one of the most commonly used tools for evaluation of stool consistency. ^1^ The BSS was developed in the early 1990s as a surrogate marker for whole-gut transit time, but is now a commonly recommended tool in clinical and research settings for the evaluation of stool consistency, rather than transit time. ^4^^,^^5^ BSS is designed to standardize the reporting of stool consistency by a 7-point ranking system, ranging from hard lumps (Type 1 on the scale) to liquid stool (Type 7 on the scale). Types 1 and 2 are considered hard stools corresponding to constipation, whereas types 6 and 7 are considered abnormally loose and watery stools corresponding to diarrhea when evaluated together with other symptoms. Types 3, 4 and 5 are generally considered “normal” stool forms.

The generalizability of BSS as a tool in research is unclear, and fecal water content or dry weight have been suggested as better objective measures. ^5^ Reporting can be inaccurate since subjects are asked to report their “average” stool form for the day, but bowel movements may vary in form throughout the day. Moreover, subjects’ interpretation of the BSS scale may vary, leading to inaccurate reporting of stool form. It is suggested that the BSS score is more precise when evaluated by a trained expert than by the patient. ^6^ Here, we aimed to evaluate the reliability of the BSS in a cohort of patients with inflammatory bowel disease (IBD), by comparing the patients’ subjective scores with scores assigned by the experienced bioengineers who received the fecal samples.

## Methods

### Design and study sample

The study is a sub-study of the IBSEN III project (Clinical Trials ID: NCT 02727959), a population-based observational inception cohort with prospective follow-up, consisting of newly diagnosed IBD patients and symptomatic non-IBD controls living in the South-Eastern Health Region of Norway, included during a 3-year period from 2017 to 2019. The study protocol for IBSEN III has been presented in detail in a previous publication. ^7^ Here, we present results from the fecal samples delivered at baseline, after 3 and 6 months and after 1 and 3 years follow-up.

### Bristol stool scale

BSS is a 7-point ranking system, where types 1-2 and 6-7 correspond to abnormal defecation with constipation and diarrhea, respectively. ^1^ For statistical analyses, the BBS score was simplified into the three categories “1 = constipation” (scores 1-2), “2 = normal” (scores 3-5) and “3 = diarrhea” (scores 6-7). This classification was chosen to correspond with the Rome IV delineation for the classification of irritable bowel syndrome (IBS) subtypes (IBS-D and IBS-C), being an applicable cut-off both in clinical practice and a research setting. ^8^

### Fecal samples

Fecal samples were collected in the IBSEN III trial from first the inclusions in 2017 and all follow-ups completed through 2022. The BSS was first implemented after a year of inclusion, hence the fecal samples included in the current analysis are those collected from January 2018 until the end of 2022. Fecal samples were delivered fresh at the hospital, after the patients had collected stool samples at home. The patients were instructed to score the stool sample with the BSS at the time of collection. Then, they were instructed to send the sample to the hospital by mail, the same day as defecation or the day after. The hospital received the sample from one to four days after defecation, and then the expert score was performed immediately (upon freezing the sample). Two experienced bioengineers analyzing the fecal samples assigned the expert score. They divided the work between them, hence the expert score is from one expert only, and not a mean value. All the fecal samples included were assessed by both an expert and a patient.

### Ethical considerations

The IBSEN III study has been reviewed and approved by the Regional Committee for Medical and Health Research Ethics in South-Eastern Norway (reference number 2015/946), and was conductance in accordance with the Declaration of Helsinki. All participants provided written informed consent.

### Statistical analysis

Categorical data are described with counts and percentages. Agreement between experts and patients was assessed using Cohen’s weighted kappa. ^9^ The results are expressed as point estimates with 95% confidence intervals (CI). P-values <0.05 were considered statistically significant. All analyses were performed using SPSS version 28 ( https://www.ibm.com/spss).

## Results

### Patient and stool sample characteristics

The study protocol and cohort’s baseline characteristics are presented in detail in a previous publication. ^7^ In total, 2970 stool samples from 1359 different patients were available for inclusion. The study subjects delivered stool samples at baseline (100%), after 3 months (48%), six months (34%), one year (26%) and three years (9%), respectively. Of the available stool samples, 2280 samples (77%) from 992 different patients had BSS scores from both patients and experts and were included in the analysis. 992 samples with matched patient and expert scores were available from baseline (100%), 513 samples from the 3-month follow-up (52% of baseline patients), 363 samples from the six-month follow-up (37% of baseline patients), 290 samples from the one-year follow-up (29% of baseline patients) and 122 samples from the three-year follow-up (12% of baseline patients).

### Patients versus expert BBS scores

**Figure 1.**
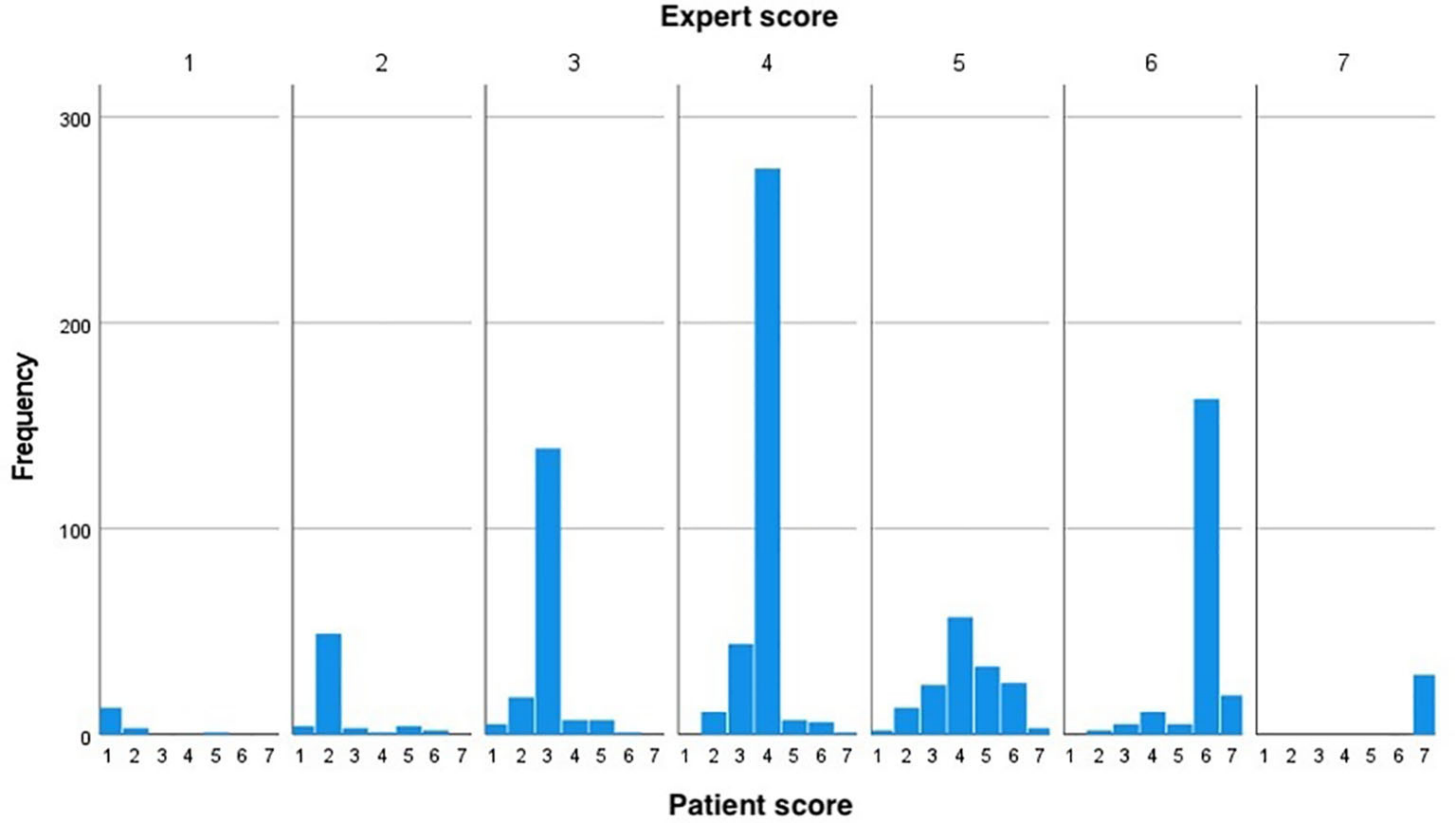
Distribution of BSS scored by patients for each value of score given by experts. The figure shows that the patients were likely to score lower than the experts. BSS: Bristol Stool Scale.

The agreement between patient and expert scores for all assessed time points was good to substantial when assessed with Cohen’s weighted kappa, ranging from 0.66 to 0.72 ( Table 1). Overall, the patients were likely to score lower than the experts. When the scores from the first sample per patient were compared between the patient and the expert ratings, the experts were more likely to assign higher scores compared to the patient’s self-assessment. The proportion of the lowest assigned scores (score 1-2) was 12.1% for patients and 8.1% for the experts. In Table 2, the scores given by the patients and experts are presented for the first assessment time point. The numbers in green represent perfect agreement between the experts and the patients. The numbers in yellow above the diagonal represent the situation when the sample is given a higher score by an expert (over-estimation) and the red numbers below the diagonal represent an under-estimation (experts score lower compared to the patients). The distribution of BSS scores reported by the patient for each value of score given by the expert is presented graphically in Figure 1.

**Table 1.** BSS score agreement between patients and experts assessed with Cohen’s weighted kappa.

Assessment time point	Original BSS scores (1-7)		Simplified BSS scores (1-3)	
	Weighted kappa	95%CI	Weighted kappa	95%CI
1 (n = 992)	0.72	0.69-0.75	0.78	0.74-0.81
2 (n = 513)	0.66	0.61-0.71	0.75	0.69-0.81
3 (n = 363)	0.71	0.66-0.76	0.73	0.66-0.80
4 (n = 290)	0.72	0.66-0.78	0.74	0.66-0.82
5 (n = 122)	0.74	0.66-0.82	0.74	0.63-0.85

BSS: Bristol Stool Scale.

**Table 2.** BSS score agreement between patients and experts at assessment point 1.

		Expert BSS scores						
		1	2	3	4	5	6	7
**Patient BSS scores**	1	13	4	5	0	2	0	0
	2	3	49	18	11	13	2	0
	3	0	3	139	44	24	5	0
	4	0	1	7	275	57	11	0
	5	1	4	7	7	33	5	0
	6	0	2	1	6	25	163	0
	7	0	0	0	1	33	19	29

BSS: Bristol Stool Scale.

The green numbers on the diagonal represent perfect agreement (the same scores) between the patients and the experts. The yellow numbers represent over-estimation, i.e. the experts scored higher than the patients. The red numbers represent under-estimation, i.e. the experts scored lower compared to the patients.

### BSS score simplified into three categories

**Figure 2.**
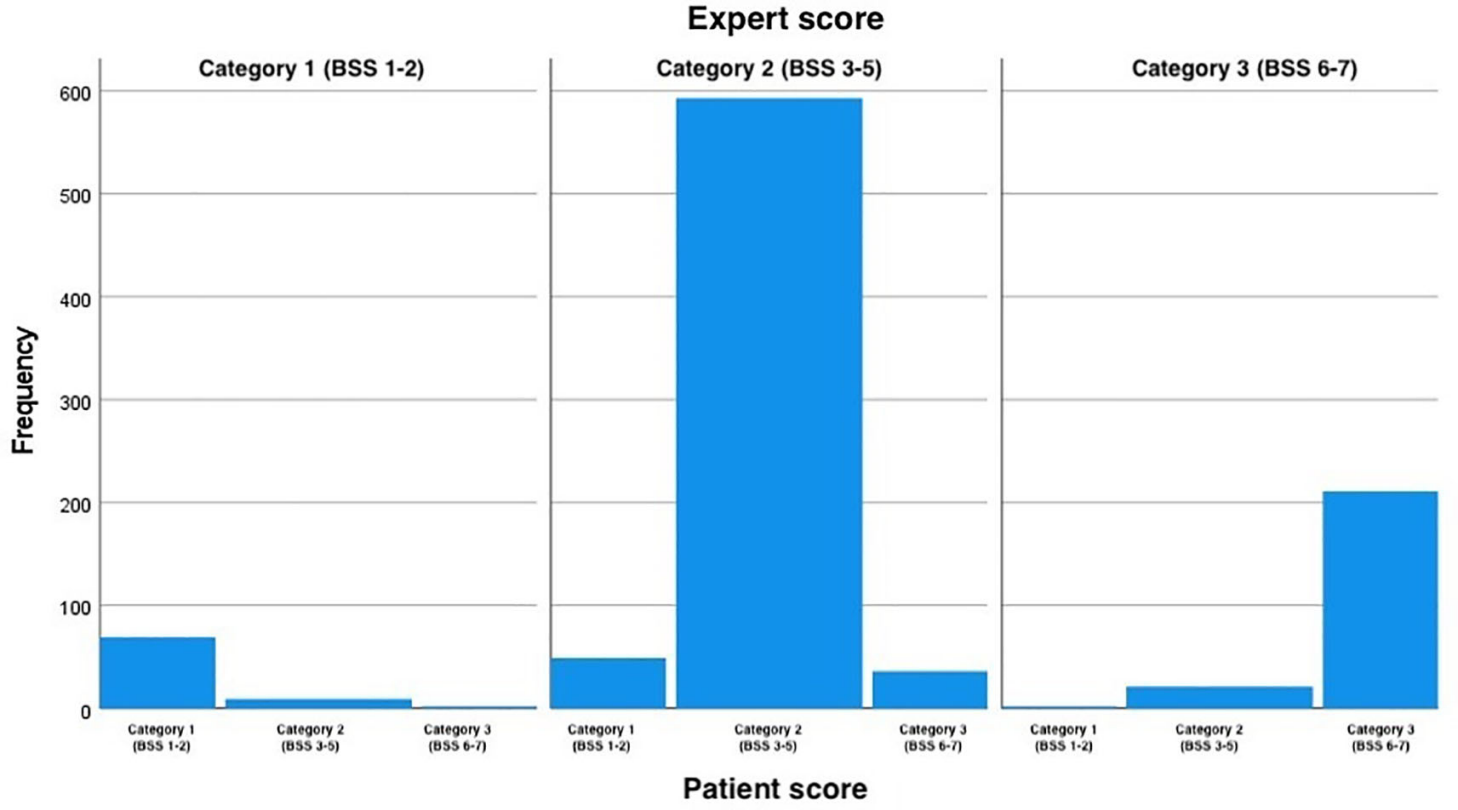
BSS scores categorized as 1, 2 and 3. Score 1 equals original scores 1 and 2 (constipation), scores 2 equals original scores 3, 4 and 5 (normal), and score 3 equals original scores 6 and 7(diarrhea). The figure shows the distribution of the BSS scored by the patient for each score value given by the expert at the first assessment point when the BSS score was simplified into the three categories. BSS: Bristol Stool Scale.

When the BSS scores were simplified and all the samples categorized as 1 (scores 1 and 2), 2 (scores 3, 4 and 5) or 3 (scores 6 and 7), patient and expert agreement improved slightly, with Cohen’s weighted kappa ranging from 0.73 to 0.77, indicating substantial agreement for all the assessed time points ( Table 1). The distribution of all scores given by the patients and experts with the simplified scores and assessment point 1 are presented in Table 3. A perfect agreement between the experts and the patients is highlighted in green, the portion of the table highlighted in yellow represents over-estimation by the experts and the red number represents an under-estimation by the experts. The distribution of the BSS scored by the patient for each value of score given by the expert at the first assessment point when the BSS score was simplified into three categories is depicted in Figure 2.

**Table 3.** BSS score agreement between patients and experts at assessment point 1 using a simplified score.

		Expert BSS scores		
		1	2	3
**Patient BSS scores**	1	69	49	2
	2	9	593	21
	3	2	36	211

BSS: Bristol Stool Scale.

The 3-category score is as follows: 1=diarrhea; 2=normal, 3=constipation.

## Discussion

BSS is commonly used in both clinical and research settings for the evaluation of stool consistency, however its reliability is unclear as the scores rely on subjective patient reports. Here, we show that there is good to substantial agreement between patient BSS scores and expert BSS scores when evaluating individual scores from 1-7. However, this agreement is even better when scores are compared in simplified categories corresponding to constipation (BSS score 1-2), normal stool (BSS score 3-5) and diarrhea (BSS score 6-7). Our findings highlight that the patient´s exact BSS score is in good agreement with the expert score, and that the patient score is accurate for evaluation of main stool type (constipation, normal or diarrhea). As the main stool type is commonly the one of most interest for the classification of disease, and more subtle distinctions might not be of great clinical importance, we found BSS to be a reliable tool for stool form evaluation.

Several studies have previously reported on the validity and use of BSS. Our findings contradict those of a previous study investigating the rate reproducibility of BSS by comparing BSS scores of thirty-four gastroenterology providers of 35 different stool photographs. ^10^ Here, they reported high reliability and agreement when BSS scores were used to assess the individual stool type from 1-7. When the experts should categorized the stool type into the three categories constipation, normal and diarrhea (corresponding to the Rome III standard ^8^), the reliability and agreement decreased. ^10^ Of note, in contrast to these prior results, our simplified into three groups was performed based on the original BSS scores and not independently assessed, hence this might explain the different findings. Also, our study compared subjective patient scores to expert scores, which is relevant as the BSS scoring is most often performed by the patients themselves.

A recent study from 2022 comparing subjective IBS patients´ BSS scores with stool water content as an objective measure reported only modest conformity between methods, highlighting that this can affect the classification of IBS-subtype according to BSS score. ^11^ Similarly, a study from 2016 validated the BSS by measuring stool form in 169 healthy adults and comparing it to stool water content and BSS score form 19 patients with diarrhea-predominant IBS. ^6^ They reported the BSS to demonstrate adequate validity and reliability, however they detected difficulties around clinical decision points for types 2, 3, 5 and 6. ^6^ Although the authors report the BSS to be a valid tool for stool evaluation, it has to be taken into consideration that the uncertainty around scorings affects the classification of stool type into constipation, normal or diarrhea, potentially affecting the clinical outcomes and diagnosis of IBS-subtype.

According to current literature highly variable results are thus presented for the validity of the BSS when comparing subjective measures with expert measures or fecal water content. BSS is widely used both in clinical and research settings, and is currently used in an increasing number of studies analyzing microbiota to adjust for differences in fecal consistency. ^5^ The unclear generalizability of BSS represents a challenge to interpreting such results. Interestingly, a recent study comparing a smartphone application using artificial intelligence (AI) to BSS scores performed by two experts reported high accuracy between the AI and the experts. In addition, they reported the trained AI to be superior to subject self-reported BSS scores, emphasising that AI assessments could provide more objective outcome measures for stool characterization in gastroenterology. ^12^ This should be taken into consideration in further studies, as it highlights a tool that can potentially improve the lack of reliability related to the subjective measures used in BSS today.

Our study has some limitations. As the follow-up period for the IBSEN III cohort is still not completed, we did not include stool samples from all subjects at all time points, and some patients were included with several samples, whereas others were only included with one sample. Due to the covid-19 pandemic, the 1-year follow-up was delayed for some of the subjects, hence the proportion of included study subjects with more than three stool samples is small. As the BSS is a subjective measure, the uneven distribution of the number of samples per participant might be a weakness affecting the results. When performing a stool sample, only a small part of the whole stool delivery is sampled for analysis. It is likely that parts of the discharge may have different consistencies and appearance, hence the part sampled for analysis might not be representative of the whole stool. This might be of particular importance when evaluating patients with GI disorders such as IBD and IBS, as the stool might be a mix ranging from hard lumps at first followed by looser stools. Our study also only included two experts. It must be considered that a larger number of experts, or a mean of several expert scores, could have strengthened the results.

## Conclusion

Taken together, our findings show that the agreement between patient and expert BSS scores is good, especially when divided into three main stool categories. We found the BSS to be a reliable tool for the categorization of stool type when the BSS scores are categorized into three categories corresponding to the clinically relevant concepts of constipation, normal stool or diarrhea.

### Ethical approval and consent

The IBSEN III study has been reviewed and approved by the Regional Committee for Medical and Health Research Ethics in South-Eastern Norway (reference number 2015/946, approval date 1 ^st^ July 2015) and was conductance in accordance with the Declaration of Helsinki. All participants provided written informed consent.

## Author contributions

HFD, JV, GHM and JTF planned and designed the study. MLH and VK included patients in the IBSEN III trial. GHM and JTF analysed the fecal samples and performed the expert BSS scoring. MCS performed the statistical analyses. HFD wrote the manuscript. JV, MCS, GHM and JTF contributed to data interpretation and critical revision of the manuscript. All authors reviewed and approved the final manuscript.

## Data Availability

Dryad: Bristol stool scale: patient versus expert score data, https://doi.org/10.5061/dryad.djh9w0w7r. ^13^ This project contains the following underlying data:
-
BSS_data_deidentified.xlsx - README.md Data are available under the terms of CC0 1.0 Universal (CC0 1.0) Public Domain Dedication license.

## References

[1] Lewis SJ Heaton KW : Stool form scale as a useful guide to intestinal transit time. *Scand. J. Gastroenterol.* Sep 1997;32(9):920–924. 10.3109/00365529709011203 9299672

[2] Törnblom H Van Oudenhove L Sadik R : Colonic transit time and IBS symptoms: what’s the link? *Am. J. Gastroenterol.* May 2012;107(5):754–760. 10.1038/ajg.2012.5 22334251

[3] Bliss DZ Savik K Jung H : Comparison of subjective classification of stool consistency and stool water content. *J. Wound Ostomy Continence Nurs.* May 1999;26(3):137–141. 10.1016/s1071-5754(99)90031-1 10711123

[4] Heaton KW O’Donnell LJ : An office guide to whole-gut transit time. Patients’ recollection of their stool form. *J. Clin. Gastroenterol.* Jul 1994;19(1):28–30. 10.1097/00004836-199407000-00008 7930429

[5] Vork L Wilms E Penders J : Stool Consistency: Looking Beyond the Bristol Stool Form Scale. *J. Neurogastroenterol. Motil.* Oct 30 2019;25(4):625. 10.5056/jnm19086 31587553 PMC6786442

[6] Blake MR Raker JM Whelan K : Validity and reliability of the Bristol Stool Form Scale in healthy adults and patients with diarrhoea-predominant irritable bowel syndrome. *Aliment. Pharmacol. Ther.* Oct 2016;44(7):693–703. 10.1111/apt.13746 27492648

[7] Kristensen VA Opheim R Perminow G : Inflammatory bowel disease in South-Eastern Norway III (IBSEN III): a new population-based inception cohort study from South-Eastern Norway. *Scand. J. Gastroenterol.* Aug 2021;56(8):899–905. 10.1080/00365521.2021.1922746 34154494

[8] Mearin F Lacy BE Chang L : Bowel Disorders. *Gastroenterology.* Feb 18 2016;150:1393–1407.e5. 10.1053/j.gastro.2016.02.031 27144627

[9] Landis JR Koch GG : The measurement of observer agreement for categorical data. *Biometrics.* Mar 1977;33(1):159–174. 10.2307/2529310 843571

[10] Chumpitazi BP Self MM Czyzewski DI : Bristol Stool Form Scale reliability and agreement decreases when determining Rome III stool form designations. *Neurogastroenterol. Motil.* Mar 2016;28(3):443–448. 10.1111/nmo.12738 26690980 PMC4760857

[11] Nordin E Hellström PM Brunius C : Modest conformity between self-reporting of Bristol stool form and fecal consistency measured by stool water content in irritable bowel syndrome, a FODMAP and gluten trial. *Am. J. Gastroenterol.* Aug 12 2022;117:1668–1674. 10.14309/ajg.0000000000001942 36087104

[12] Pimentel M Mathur R Wang J : A Smartphone Application Using Artificial Intelligence Is Superior to Subject Self-Reporting When Assessing Stool Form. *Am. J. Gastroenterol.* Mar 14 2022;117:1118–1124. 10.14309/ajg.0000000000001723 35288511

[13] Valeur J : Data from: Bristol stool scale: Patient versus expert score data.[Dataset]. *Dryad.* 2024. 10.5061/dryad.djh9w0w7r

