# Supplementary material for: Assessing hard and loose “endpoints”: comparison of patient and expert Bristol Stool Scale scoring of 2280 fecal samples
Source: F1000Res. 2024 Dec 19;13:833. Originally published 2024 Jul 25. [Version 2] doi: 10.12688/f1000research.152496.2 (PMC11770634; doi:10.12688/f1000research.152496.2)
Supplement: Peer review history

# Reviewer report: Reviewer response for version 2

**DOI:** 10.5256/f1000research.175975.r350717

**Published:** 2024-12-24

**Authors:** Friedemann Erchinger

**Affiliations:** 1 University of Bergen, Bergen, Norway

No further comments.

Is the work clearly and accurately presented and does it cite the current literature?

Yes

If applicable, is the statistical analysis and its interpretation appropriate?

Yes

Are all the source data underlying the results available to ensure full reproducibility?

Yes

Is the study design appropriate and is the work technically sound?

Yes

Are the conclusions drawn adequately supported by the results?

Yes

Are sufficient details of methods and analysis provided to allow replication by others?

Yes

Reviewer Expertise:

exocrine pancreatic insufficiency, fecal fat, fecal output

I confirm that I have read this submission and believe that I have an appropriate level of expertise to confirm that it is of an acceptable scientific standard.

# Reviewer report: Reviewer response for version 1

**DOI:** 10.5256/f1000research.167263.r344492

**Published:** 2024-12-11

**Authors:** Friedemann Erchinger

**Affiliations:** 1 University of Bergen, Bergen, Norway

The article describes agreement between patients and experts when scoring stool consistency after the Bristol Stool Scale.

 Using Cohen’s weighted kappa for all scales 1 to 7 or grouped scales, constipation (1-2) normal (3-5) and diare (6,7), agreement was substantial; grouped scales show an even better agreement.

 To use easy methods which can be practiced by patients and experts is a needed tool in a clinical setting and in research. This makes this study important.

 Not all authors relate BSS value 5 to normal consistency. Some categorise value 5 as  "Borderline" (normal/diarrhoea), others as diarrhoea. Perhaps this should be discussed in the article.

 It would have been attractive to show interobserver and intraobserver variability in BSS scoring, both in patients (with relatives as interobservers?) and experts. This would have strengthened the study substantially. Take this point in in "limitations"?

 As discussed can stool frequency give different stool consistency. Perhaps agreement got better if experts knew about stool frequency?

 Discuss this point in "limitations"?

 As discussed is stool delivered in one session not homogeneous, stool samples mailed to scoring can contain only a part of the entire stool delivery.

 -This could be overcome by photographs of the stools. Discuss this in "future considerations"?

 -As patients look at the entire stool delivery and experts only at a small sample it is very interesting that agreement can be classified as substantial. This should be highlighted in the article.

Is the work clearly and accurately presented and does it cite the current literature?

Yes

If applicable, is the statistical analysis and its interpretation appropriate?

Yes

Are all the source data underlying the results available to ensure full reproducibility?

Yes

Is the study design appropriate and is the work technically sound?

Yes

Are the conclusions drawn adequately supported by the results?

Yes

Are sufficient details of methods and analysis provided to allow replication by others?

Yes

Reviewer Expertise:

exocrine pancreatic insufficiency, fecal fat, fecal output

I confirm that I have read this submission and believe that I have an appropriate level of expertise to confirm that it is of an acceptable scientific standard, however I have significant reservations, as outlined above.

# Response

**Published:** 2024-12-13

**Authors:** Jørgen Valeur

**Affiliations:** Not Applicable, Norway

We would like to thank the Reviewer for taking the time to read and comment on our paper, and are grateful for both the encouraging comments, as well as the valuable suggestions.

 Our point-by-point response is as follows:

 1. " *Not all authors relate BSS value 5 to normal consistency. Some categorise value 5 as  "Borderline" (normal/diarrhoea), others as diarrhoea. Perhaps this should be discussed in the article."*

**Response**: We agree, and have now included this sentence in Methods: *"It should be noted, however, that some authors consider a score of 5 as abnormal."*

 2. *"It would have been attractive to show interobserver and intraobserver variability in BSS scoring, both in patients (with relatives as interobservers?) and experts. This would have strengthened the study substantially. Take this point in in "limitations"?"*

**Response**: We agree that this would have been valuable information to include, and have now taken this point in in "limitations", as suggested: *"Similarly, including an assessment of interobserver and intraobserver variabilities, both in the patient group and among the experts, would have strenghtened our study substantially."*

 3. *"As discussed can stool frequency give different stool consistency. Perhaps agreement got better if experts knew about stool frequency? Discuss this point in "limitations"?"*

**Response:** We agree that it would have been valuable to know the stool frequencies of the participants. We have now included this sentence in "limitations": *"It would have been interesting to know the participants stool frequency, since the frequency may affect the consistency."*

 4. *"As discussed is stool delivered in one session not homogeneous, stool samples mailed to scoring can contain only a part of the entire stool delivery.*

*-This could be overcome by photographs of the stools. Discuss this in "future considerations"?*

*-As patients look at the entire stool delivery and experts only at a small sample it is very interesting that agreement can be classified as substantial. This should be highlighted in the article."*

**Response:** We would like to thank the Reviewer for pointing this out, and have now included the following sentence in Conclusion: *"Indeed, our finding that expert scoring of only a small fecal sample is in good agreement with the patient scoring of the entire stool delivery is a novel observation that should be validated in future studies where photographs of the entire stool delivery should also be included."*

 Althogether, we now sincerely hope that the Reviewer may find our paper to deserve the status as "Approved".

# Reviewer report: Reviewer response for version 1

**DOI:** 10.5256/f1000research.167263.r315298

**Published:** 2024-08-24

**Authors:** Bodil Ohlsson

**Affiliations:** 1 Clinical Sciences, Lund University, Malmo, Sweden

The study aimed to compare the assessment of coherence between the patients and the experts scores regarding Bristol Stool Scale (BSS). 2280 fecal samples collected from 992 patients with inflammatory bowel disease (IBD) at up to five time points for 3 years were included. Agreement between patient and expert scores was assessed using Cohen’s kappa.

 There was good substantial agreement between patient and expert scores (Cohen’s weighted kappa: 0.66-0.72). When the BSS scores were simplified and categorized into 3 groups instead of 7, the agreement improved slightly (Cohen’s weighted kappa: 0.73-0.77).

 To summarize, the agreement between patient and expert BSS scores is good to substantial, especially when the BSS scores are simplified into three categories: constipation, normal stool and diarrhea.

 Remarks:

 In the discussion, it is stressed that these results with good coherence between patients and expert scores differ from a previous study performed in IBS. I think that IBS and IBD patients differ a lot. IBS patients may have greater psychological influence on their symptoms and may interpret their symptoms in other ways than IBS patients with more obvious organic changes. The difference between these 2 patient cohorts and psychological aspects regarding self-reported assessment should be discussed.

Is the work clearly and accurately presented and does it cite the current literature?

Yes

If applicable, is the statistical analysis and its interpretation appropriate?

Yes

Are all the source data underlying the results available to ensure full reproducibility?

Yes

Is the study design appropriate and is the work technically sound?

Yes

Are the conclusions drawn adequately supported by the results?

Partly

Are sufficient details of methods and analysis provided to allow replication by others?

Yes

Reviewer Expertise:

Gastroenterology

I confirm that I have read this submission and believe that I have an appropriate level of expertise to confirm that it is of an acceptable scientific standard.

# Response

**Published:** 2024-12-13

**Authors:** Jørgen Valeur

**Affiliations:** Not Applicable, Norway

We would like to thank the Reviewer for taking the time to read and comment on our paper, and are grateful for both the encouraging comments, as well as the valuable suggestions. In our revised version, we have now included the following sentence in Discussion: *"It should be emphasized that self-reported assessments may differ between IBS patients and IBD patients, since psychological factors may have a greater influence of IBS symptoms than IBD symptoms."*
